# Extensive Gastric Necrosis and Perforation Secondary to Intragastric Balloon-Related Gastric Outlet Obstruction: A Case Report

**DOI:** 10.7759/cureus.116444

**Published:** 2026-09-17

**Authors:** Abderrahim Ait Abderrhim, Hicham Assofi, Karim Ibn Majdoub Hassani

**Affiliations:** 1 Department of Visceral Surgery, University Hospital Center Hassan II, Fez, MAR

**Keywords:** gastric necrosis, gastric outlet obstruction, gastric perforation, intragastric balloon, total gastrectomy

## Abstract

Intragastric balloons (IGBs) are widely used as temporary endoscopic therapy for obesity, but rare complications can be catastrophic. A 55-year-old woman presented with severe epigastric pain, persistent vomiting, and progressive abdominal distension four months after the placement of a 650-mL Orbera intragastric balloon (Apollo Endosurgery, Austin, TX). She was hypotensive and tachycardic and presented with generalized peritonitis. Contrast-enhanced CT revealed marked gastric distension, antropyloric balloon impaction, absent mural enhancement along the lesser curvature, pneumoperitoneum, and free intraperitoneal fluid. Emergency laparotomy uncovered extensive gastric necrosis with a 4-cm perforation. The intact balloon was removed, and total gastrectomy without immediate esophagojejunal reconstruction was performed along with feeding jejunostomy. Histopathological examination confirmed complete transmural infarction and necrosis of the lesser curvature, severe diffuse acute ischemic injury of the adjacent gastric wall with vascular microthrombi, and localized fibrinous peritonitis, with no malignant neoplasm. Despite intensive care, refractory septic shock and multiorgan failure continued to progress, and the patient died 72 hours postoperatively. This case highlights the need for urgent imaging and source control when persistent pain, vomiting, or distension develops in a patient with an IGB.

## Introduction

Intragastric balloons (IGBs) are temporary endoscopic devices that can achieve clinically meaningful weight loss, and serious adverse events remain uncommon [1,2]. Gastric outlet obstruction, ulceration, ischemia, and perforation are rare but potentially fatal complications [2,3]. Published cases have described gastric necrosis or ischemic perforation after IGB placement [4-6]. Other focal perforations have been managed with primary repair or wedge resection [7,8]. We describe a case of extensive gastric necrosis and perforation secondary to IGB-related gastric outlet obstruction, which required emergency total gastrectomy and a staged damage-control strategy.

## Case presentation

A 55-year-old woman with hypertension and obesity (BMI: 38.5 kg/m²) had undergone endoscopic placement of a 650-mL saline-filled Orbera IGB (Apollo Endosurgery, Austin, TX) at another institution. A surveillance endoscopy performed two months later showed an intact, well-positioned balloon with no mucosal erosion. Four months after the procedure, she developed severe epigastric pain, repeated vomiting, and progressive abdominal distension. Her symptoms had been ongoing for approximately 24 hours before admission. Upon arrival, her blood pressure was 90/60 mmHg, her heart rate was 118 beats/min, her respiratory rate was 26 breaths/min, and her temperature was 38.4°C. Her abdomen was markedly distended, with generalized guarding and rebound tenderness present.

Admission laboratory findings are summarized in Table 1. These results demonstrated marked leukocytosis and inflammation, acute kidney injury, acidemia, and hyperlactatemia, findings consistent with septic shock and tissue hypoperfusion.

**Table 1:** Admission laboratory findings

Laboratory parameter	Patient value	Reference value
White blood cell count	18,500/mm³	4,000-11,000/mm³
C-reactive protein	287 mg/L	< 10 mg/L
Serum creatinine	1.8 mg/dL	0.6-1.1 mg/dL
Arterial pH	7.28	7.35-7.45
Serum lactate	5.8 mmol/L	< 2.0 mmol/L

Intravenous fluid resuscitation, broad-spectrum antibiotics, and vasopressor support were all initiated. A contrast-enhanced CT scan was performed 75 minutes after admission. It revealed massive gastric distension and a fluid-filled balloon impacted in the antropyloric region, which was causing gastric outlet obstruction. The absence of mural enhancement along the lesser curvature, along with free intraperitoneal air and free fluid, was consistent with gastric ischemia and perforation. Figure 1 illustrates the marked gastric distension and the distal position of the balloon. The celiac trunk and superior mesenteric artery were reported to be patent, with no evidence of mesenteric venous thrombosis.

**Figure 1:**
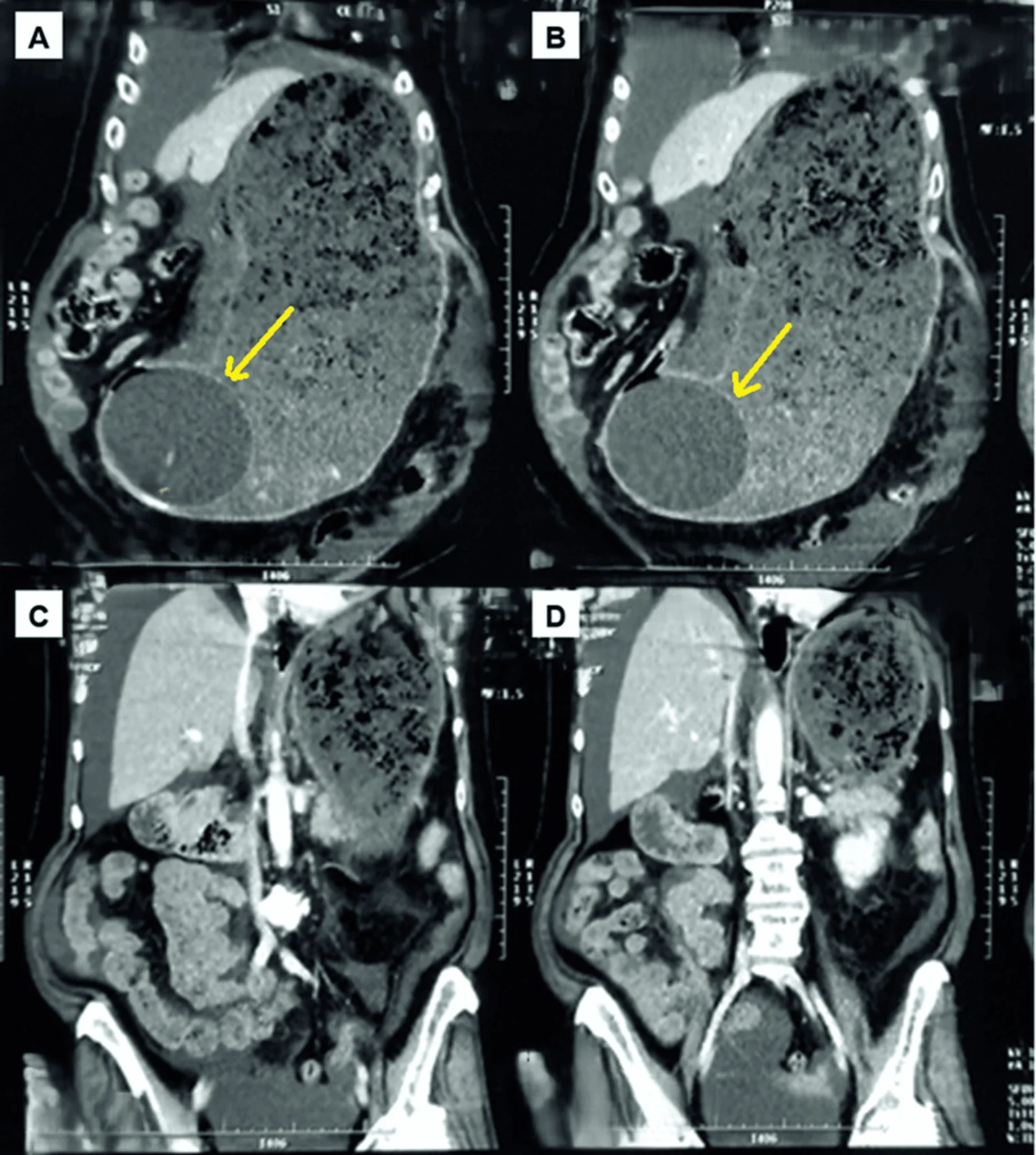
Contrast-enhanced CT findings Coronal contrast-enhanced CT images demonstrate massive gastric distension with heterogeneous intraluminal contents. The fluid-filled intragastric balloon is visible in the distal stomach (yellow arrows in panels A and B). Panels C and D further demonstrate the marked extent of gastric distension CT: computed tomography

An emergency midline laparotomy was begun 165 minutes after admission and 90 minutes after the CT scan. Exploration revealed generalized contamination by gastric contents and extensive necrosis involving approximately 70% of the gastric surface, predominantly affecting the entire lesser curvature, substantial portions of the fundus, and the anterior gastric body. A 4-cm perforation was found on the lesser curvature, approximately 8 cm distal to the gastroesophageal junction, its margins necrotic and friable.

The balloon was removed intact (Figure 2). Macroscopic examination revealed an intact silicone envelope and filling valve, with no visible tears, leakage, gross deflation, or deformation. Its volume was estimated to be approximately 650 mL, although no formal post-extraction volume measurement was documented.

**Figure 2:**
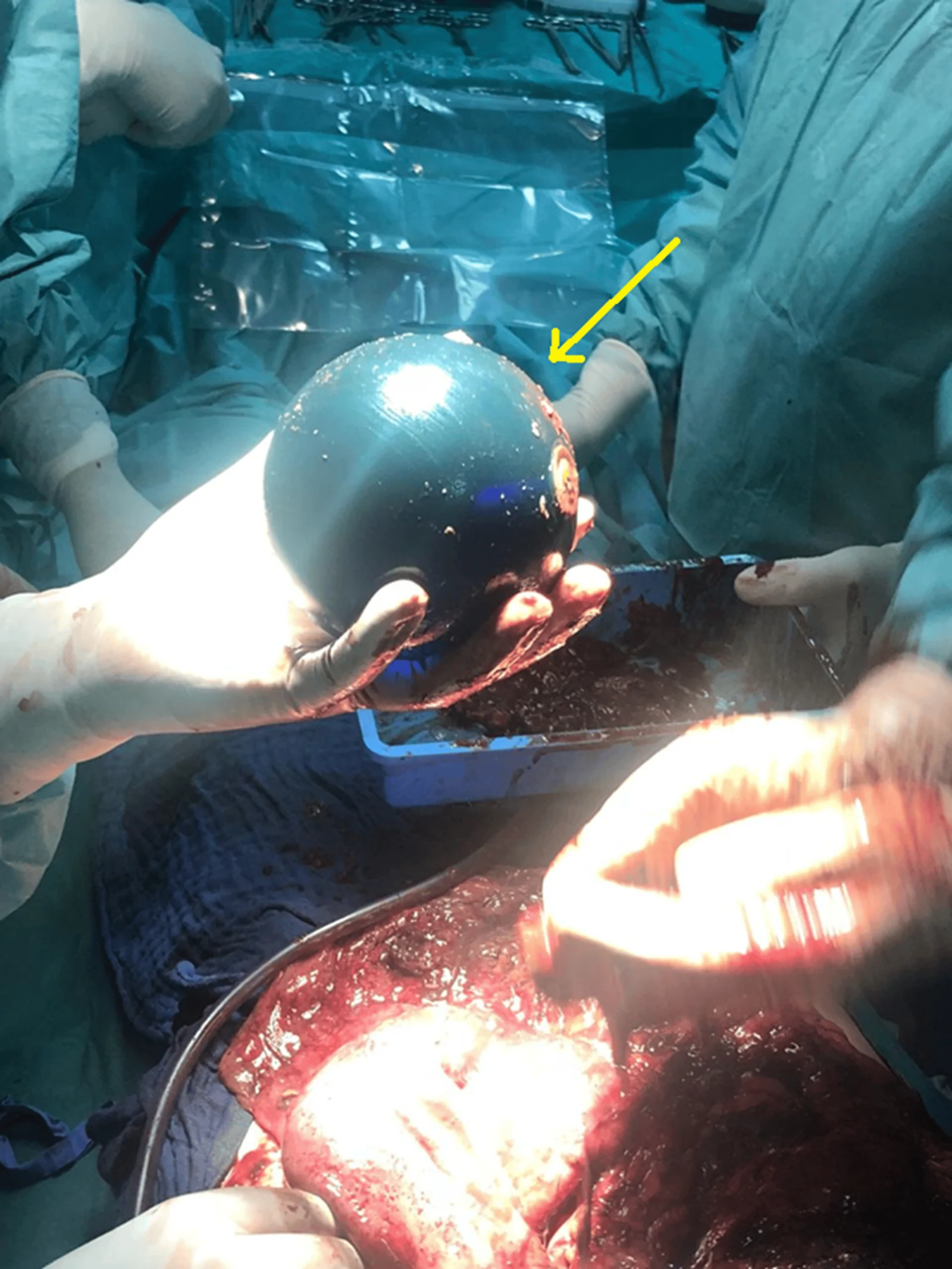
Intraoperative removal of the intragastric balloon Intraoperative photograph showing removal of the intact fluid-filled Orbera intragastric balloon (yellow arrow) after gastric decompression

After balloon extraction and gastric decompression, the posterior gastric body and antrum were still severely ischemic, while the greater curvature was markedly congested and edematous. Only a small area was considered potentially viable. Major gastric arterial pulsations were noted intraoperatively, although this did not confirm adequate mural perfusion. The extent and distribution of tissue injury left no gastric remnant sufficiently viable for preservation; a total, rather than subtotal, gastrectomy was therefore performed (Figure 3). Immediate esophagojejunal reconstruction was deferred, and the esophageal and duodenal ends were closed, with a feeding jejunostomy created as part of a staged damage-control strategy.

**Figure 3:**
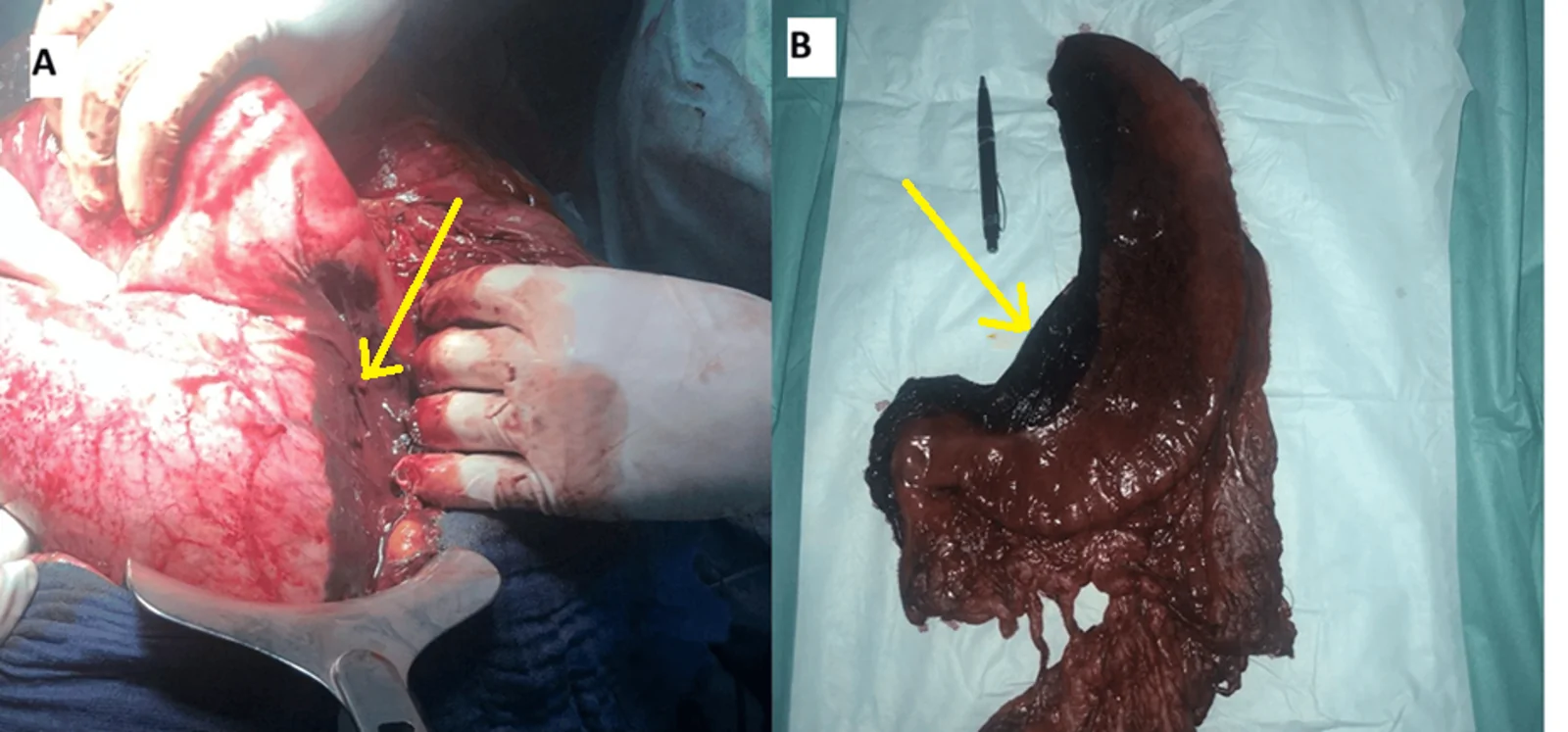
Operative gastric necrosis and total gastrectomy specimen (A) Intraoperative view showing extensive dark, dusky gastric wall consistent with nonviable tissue (yellow arrow). (B) Total gastrectomy specimen showing widespread dark discoloration of the gastric wall (yellow arrow)

Postoperatively, refractory septic shock continued to progress despite mechanical ventilation, renal replacement therapy, broad-spectrum antimicrobial therapy, and multiple vasopressors. Early postoperative laboratory findings included an arterial pH of 7.22, a serum lactate of 6.8 mmol/L, a serum creatinine of 2.4 mg/dL, and an INR of 1.6. The patient subsequently developed multiorgan failure and died 72 hours after surgery. Histopathologic examination of the gastrectomy specimen revealed complete transmural (panmural) infarction and necrosis of the lesser curvature, complicated by localized fibrinous peritonitis. The adjacent gastric wall showed severe, diffuse, acute ischemic injury with vascular microthrombi. No malignant neoplasm was identified.

## Discussion

Gastric necrosis after IGB placement is exceptionally rare. Proposed mechanisms include focal pressure injury, ulceration, balloon hyperinflation, and obstruction [2-5,9]. In this patient, balloon impaction at the gastric outlet, marked upstream distension, and extensive ischemic injury all support an obstruction-related mechanism. Pressure-related impairment of intramural perfusion is plausible, but it was not directly measured. Although major arterial patency was reported on CT and arterial pulsations were noted intraoperatively, these findings do not rule out microvascular thrombosis or nonocclusive ischemia. Hypovolemia, septic shock, and associated coagulation abnormalities may also have been contributing factors.

Histopathology confirmed transmural infarction and vascular microthrombi, but it could not establish whether the thrombosis preceded or followed the ischemic injury. Other possible mechanical contributors include focal balloon-related pressure injury, interval ulceration, and hyperinflation. No volvulus was described in the available imaging or operative report. The normal surveillance endoscopy two months earlier does not rule out subsequent ulceration, and the absence of a formally measured post-extraction balloon volume means hyperinflation cannot be definitively excluded. As a result, the precise causal sequence remains uncertain.

The distinctive feature of this case is the combination of complete gastric outlet obstruction, extensive transmural necrosis, and perforation, occurring despite a normal surveillance endoscopy two months earlier. Previous reports have described ischemic injury or delayed perforation, while extensive necrosis requiring gastrectomy appears to be particularly uncommon [4-8]. Contrast-enhanced CT is central to the evaluation of severe or persistent symptoms, because it can identify obstruction, mural nonenhancement, free air, and the extent of intraperitoneal contamination [9,10].

Localized necrosis or perforation may be treatable with primary repair or wedge resection [6-8]. When gastric tissue is extensively nonviable and accompanied by shock, definitive resection may be necessary. In this case, deferring reconstruction shortened the index operation and avoided a high-risk anastomosis during a period of profound physiological derangement. The fatal outcome illustrates how the progression from obstruction and ischemia to perforation, septic shock, and multiorgan failure can occur rapidly once persistent pain and vomiting begin.

This report has several limitations. The precise duration of complete obstruction before presentation could not able to be determined, and no direct measurement of gastric perfusion or dedicated vascular imaging was available. Although histopathology confirmed transmural infarction, acute ischemic injury, and vascular microthrombi, these findings do not clarify whether the microthrombosis preceded or followed the ischemic injury. Therefore, the proposed pressure-related mechanism is supported by the combined radiological, operative, and pathological findings but cannot be definitively proven.

## Conclusions

Severe pain, persistent vomiting, or progressive abdominal distension in a patient with an IGB should not be automatically attributed to expected device intolerance. Urgent contrast-enhanced CT and early multidisciplinary source control are warranted when obstruction, ischemia, or perforation are suspected. Extensive gastric nonviability may require total gastrectomy and staged reconstruction. The prognosis is poor once advanced septic shock and multiorgan failure have developed.
